# Immune-Mediated Necrotizing Myopathy, Associated With Antibodies to Signal Recognition Particle, Together With Lupus Nephritis: Case Presentation and Management

**DOI:** 10.14740/jocmr2133w

**Published:** 2015-04-08

**Authors:** John O’Grady, Len Harty, Nick Mayer, Val Critcher, John Ryan

**Affiliations:** a Department of Rheumatology, Cork University Hospital, Wilton, Cork City, Cork, Ireland; b Cork University Hospital, Wilton, Cork City, Cork, Ireland

**Keywords:** Immune, Necrotizing myopathy, Signal recognition particle, Lupus nephritis

## Abstract

A male patient with limb weakness, myalgia and edema was subsequently found to have an immune-mediated necrotizing myopathy (IMNM) on biopsy. Targeted myopathic antibody analysis revealed antibodies to signal recognition particle (SRP). Anti-SRP-associated necrotizing myopathy was diagnosed. This case was complicated by the concurrent development of class III lupus nephritis. We discuss an interesting case progression and development as well as the management of these difficult to treat conditions.

## Introduction

Treatment can prove challenging for signal recognition particle (SRP)-associated, immune-mediated myopathy. In our case, this was made all the more difficult by development of lupus nephritis, class III. However, the presence of anti-SRP antibodies can help guide therapy in difficult to treat myopathies, necessitating the need for plasmapheresis. Here, we detail the progression of such a case and outline management strategies to achieve remission.

## Case Report

A 52-year-old male presented with bilateral lower limb cramping pains, myalgia and increasing weakness of proximal musculature of both upper and lower limbs.

### History and exam

A 4-week history of these symptoms was associated with generalized fatigue, malaise and anorexia. The onset of symptoms was gradual and progressive over this period. At the time of presentation, bilateral lower limb pitting edema became evident, which was not present previously. There was no evidence of cutaneous rash, ocular symptoms, oral ulceration, arthralgias or nail changes. There was no dysphagia or other gastrointestinal symptoms. Systems review was non-contributory from a cardiac and respiratory perspective.

There was no history of foreign travel, no noted exposure to contractible diseases or recent infectious symptoms.

Past medical history was noted for hypercholesterolemia and hypertension. Medications included an ACE inhibitor and statin therapy.

This gentleman worked as a builder in his local community. He did not consume alcohol. He smoked 30 cigarettes per day for 40 years, equivalent to 60 pack-years. He was previously fully independent of all activities.

Clinical examination revealed bilateral upper and lower limb proximal muscle weakness, with a clinical power grade of two out of five. Lower limb bilateral edema was also evident. Vital statistics were within normal range apart from a resting tachycardia of 110 beats/min. Examination was otherwise unremarkable.

### Initial laboratory investigations

Hematology shows normal full blood count and elevated ESR of 111 mm/h.

Biochemistry shows normal urea, creatinine and electrolytes apart from sodium, decreased at 124 mmol/L. Deranged liver enzymes were noted: AST 2,131 IU/L, ALT 593 IU/L, ALP 194 IU/L, GGT 205 IU/L, bilirubin 8 mg/L, and albumin 19 g/L. Alpha fetoprotein was normal. LDH markedly elevated at 4,335 IU/L. Creatine kinase levels recorded as 20,000 IU/L. Thyroid function testing was normal and corrected calcium was also normal. Serum protein electrophoresis was negative for a paraprotein band.

ANA was strongly positive, homogenous. Double-stranded DNA titer level was 77 IU/mL, with negative crithidia dsDNA. Complement C3 and C4 levels were 0.57 mg/L and 0.08 mg/L respectively. IgM rheumatoid factor, anti-CCP antibody and anti-neutrophil cytoplasmic antibody levels were all within normal range.

Screening tests for cytomegalovirus, Epstein-Barr virus, toxoplasmosis, parvovirus B19 and hepatitis B and C virus were negative.

Urine dipstick displayed 2+ proteinuria, with a 24-h protein collection of 7 g.

### Working diagnosis and management

Presentation and initial investigations were highly suggestive of myositis and of nephrotic syndrome. A unifying working diagnosis of systemic lupus erythematosus (SLE) with lupus nephritis was made. Initial treatment with oral prednisolone proved ineffective. Mycophenolate mofetil and intravenous methylprednisolone were commenced with subsequent oral changeover to high dose prednisolone at 1 mg/kg. No discernible clinical benefit was obtained with this therapy.

### Investigations and hospital course

Kidney needle biopsy appearances were in keeping with focal lupus nephritis class III (A/C) (International Society of Nephrology/Renal Pathology Society ISN/RPS 2003 classification). Glomeruli showed diffuse global mesangial matrix expansion and hypercellularity with scattered leukocytes and at least one glomerulus showed active lesions characterized by segmental endocapillary proliferation. Capillary wall and mesangial IgG, IgM, IgA, C3 and C1q were positive on immunofluorescense. Electron microscopy confirmed small, subendothelial, mesangial and paramesangial immune-type deposits.

**Figure 1:**
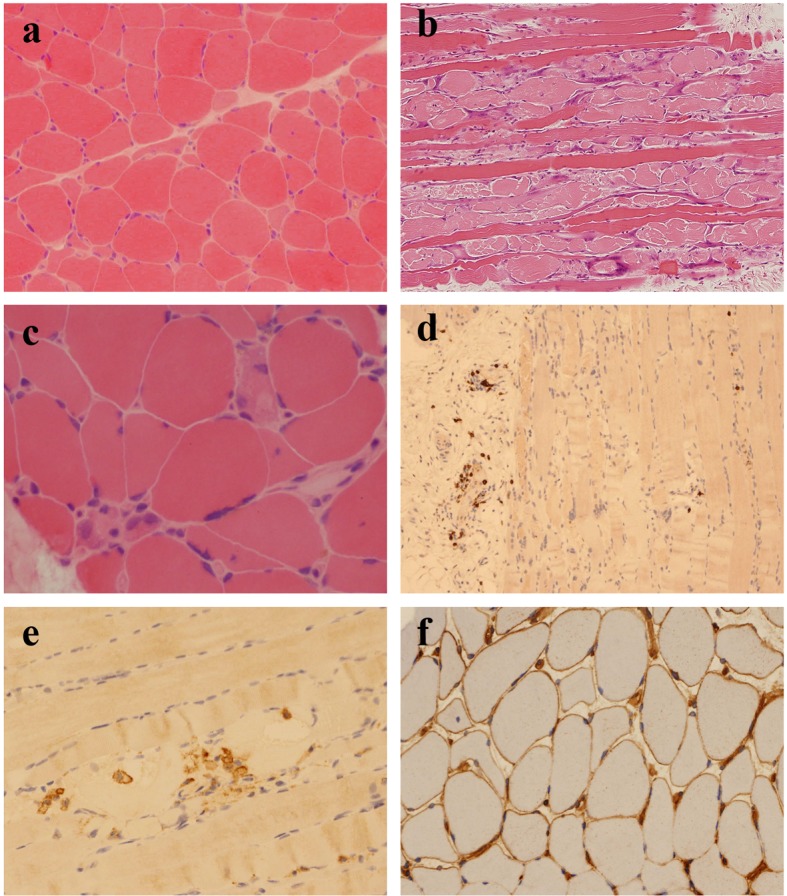
(a) Scattered, angulated and rounded, atrophic fibers (H&E, cryostat section). (b) Large numbers of necrotic and basophilic regenerating fibers (H&E, paraffin section). (c) Necrotic fibers occurring singly and in small groups, many showing myophagocytosis (H&E, cryostat section). (d) Small numbers of CD8^+^ T lymphocytes, mostly confined to perivascular locations (CD8 immunohistochemistry). (e) Macrophages within necrotic fibers (CD68 immunohistochemistry). (f) Patchy sarcolemmal upregulation of MHC class I antigen (MHC class I immunohistochemistry).

An open biopsy of quadriceps muscle was performed approximately 6 weeks after onset of symptoms and 2 weeks after commencement of combined mycophenolate and steroid therapy. It showed an increase in fiber size variation, with angulated and rounded atrophic fibers scattered throughout the fascicles (Fig. 1a). There was no obvious type-selective atrophy. Necrotic fibers were seen throughout the biopsy, occurring singly and in small groups, many showing myophagocytosis. There were also many basophilic regenerating fibers (Fig. 1b, c). There were scattered end-stage nuclear clusters. A mild increase in frequency of internalized nuclei was seen in normal fibers. There was a very mild, focal increase in endomysial fibrosis. Vacuolation was confined to necrotic fibers. No ragged red fibers or inclusions were seen. A single COX-negative fiber was identified at one level. No increase in accumulation of glycogen or lipid was present. Other than macrophage activity associated with the necrotic fibers, no prominent inflammatory cell infiltration was seen in the endomysium. Small collections of lymphocytes and plasma cells were seen surrounding a few of the perimysial vessels. There was no evidence of vasculitis. On immunohistochemistry, the perivascular aggregates were shown to contain B lymphocytes (CD20 positive) and T lymphocytes (CD4 and CD8-positive subsets equally represented) (Fig. 1d). A very few isolated T lymphocytes were demonstrated within the endomysial compartment. No lymphocytic infiltration of non-necrotic fibers was seen. CD68 highlighted a mild increase in macrophage activity in the endomysial and perimysial compartments, in addition to the macrophages within necrotic fibers (Fig. 1e). The MHC class I antigen preparation showed focal sarcolemmal upregulation on normal fibers, with more prominent expression on necrotic and regenerating fibers (Fig. 1f). There was deposition of membrane attack complex (MAC; C5b-9) on the necrotic fibers and very focally on endomysial capillaries. In summary the appearances were those of a pauci-inflammatory, necrotizing myopathy and they raised the possibility of an immune-mediated etiology.

Following this, targeted myopathic antibodies were sought. Antibodies to SRP on serum analysis were detected, leading to the diagnosis of anti-SRP-associated necrotizing myopathy, together with class III lupus nephritis.

### Further management

Rituximab infusions of 375 mg/m^2^ were commenced as a next line of treatment; however, clinical improvement was not achieved. Daily physiotherapy supplemented medical therapies in attempting to control and improve the clinical condition.

Following this, plasmapheresis was performed over a 3-week period, three times per week. After the third session proximal power improved clinically, to grade four, and creatine kinase was found to markedly decrease to 1,247 IU/L. No significant adverse reactions were encountered and the patient recovered with both the myopathy and lupus nephritis clinically remitting.

### Follow-up

Discontinuation of immunosuppressive agents resulted in a relapse of symptoms and signs. There was an associated increase in creatine kinase levels also. Azathioprine together with oral steroid maintenance therapy was commenced with successful clinical improvement. This phase of treatment was complicated by an acute hospital admission for treatment of *Pneumocystis jirovecii* pneumonia. The patient had been doing clinically well with azathioprine therapy and slow tapering of oral steroid dosing following this admission. The patient passed away suddenly from an unrelated cause a number of months later. He had been in clinical and serological remission from his myopathy.

## Discussion

Of patients with SLE, 4-16% display evidence of muscle involvement, most often at time of diagnosis [1, 2]. SLE, in a case with diagnosed class IV lupus nephritis concurrently, has been associated with development of intestinal myopathy [3]. Skeletal muscle involvement in SLE patients may manifest as weakness, myalgia and atrophy, often in a proximal distribution [4]. Overlap syndromes of SLE with myositis, including dermatomyositis and polymyositis, are recognized clinically [1, 2]. However, although abnormal muscle biopsies are common in patients with SLE, myositis accounts for relatively few of the changes seen and other key features, particularly type II selective fiber atrophy and lymphocytic vasculitis, may be present [4].

Antibodies to SRP can be detected by methods that use either a ribonucleic acid immune-precipitation assay or an assay involving SRP54 as an antigen for detection of antibodies [5]. The antibodies have been found in around 4-6% of patients presenting with idiopathic inflammatory myopathy (IIM) [6]. SRPs are cytoplasmic complexes of a small RNA and six SRP family proteins. Their function is to guide newly translated proteins into the endoplasmic reticulum. As SRP expression is ubiquitous, the link between anti-SRP antibodies and myopathy is uncertain [7]. On a serological basis of classification, anti-SRP-associated myopathy appears to define a distinct entity based on epidemiology, symptoms and response to treatment. In contrast to some other myositis syndromes, it does not commonly overlap with connective tissue diseases [7-11] and so the association of anti-SRP antibodies with SLE and lupus nephritis in this patient is unusual.

Rapidly progressing muscle weakness is a recognized feature of this myopathy subtype [5]. Proximal muscle groups more than distal, in both upper and lower limbs, are affected. Muscle pains, generalized fatigue and involvement of other muscle groups can occur [12]. High serum creatinine kinase levels are often seen on serum analysis [10]. Cases associated with dysphagia, cardiac involvement, interstitial lung disease and skin rash have been reported [5, 10, 13].

Whilst SRP-associated myopathy shows some clinical heterogeneity, the pathological findings seem to be more consistent [12]. Typically there is increased variation in fiber size. There are large numbers of necrotic and regenerating fibers at various stages of injury. There may be a mild increase in endomysial fibrosis. Other than macrophages associated with necrotic fibers, inflammation is sparse, particularly in the endomysial compartment, although focal collections of lymphocytes (T and B cells) may be seen around vessels. Focal invasion of intact muscle fibers by mononuclear cells is not seen. Capillary changes can overlap with features seen in dermatomyositis and may include capillary enlargement, pipestem capillaries and reduced capillary density. Deposition of C5b-9 on endomysial capillaries may be patchy or absent. Sarcolemmal upregulation of MHC class I antigen is usually absent or weak and focal but is seen on regenerating fibers [10-13].

The above features are consistent with the muscle biopsy criteria that characterize the immune-mediated necrotizing myopathies (IMNMs). These are a group of diseases within the spectrum of IIM [11, 14]. The IMNMs are associated with autoimmune antibodies other than SRP, including the antisynthetase antibodies (such as anti Jo-1) and anti-HMGCR antibodies. They also include paraneoplastic necrotizing myopathy and some connective tissue diseases. However, a much broader spectrum of disorders can give a muscle biopsy appearance of necrotizing myopathy. Clinical consideration should be given to the possibility of a toxic or drug-induced etiology. Certain genetic muscle disorders may also show fiber necrosis with focal inflammation on muscle biopsy and may clinically present with subacute proximal weakness and elevated CK levels; these include dysferlinopathy and facioscapulohumeral muscular dystrophy.

SRP-associated myopathy can pose treatment challenges. Steroid monotherapy, often ineffective in this myopathy subtype, used early in the disease course has been reported to improve muscle power [10]. Methotrexate, cyclophosphamide, ciclosporin, rituximab, immunoglobulins and plasmapheresis have all been used with variable success [6, 13, 15, 16]. Plasmapheresis in combination with either rituximab or cyclophosphamide has achieved successful remission [17, 18].

Lupus nephritis is classified into six groups. Class III, as with our patient, is defined as focal lupus nephritis that involves less than 50% of the glomeruli. The glomerular lesions consist of either active and/or inactive (sclerotic) lesions and lupus vasculopathy and tubulointerstitial nephritis are also recognized components of lupus nephritis [19].

Treatment options include corticosteroids alone, or in combination with mycophenolate mofetil, cyclophosphamide, cyclosporin, tacrolimus or rituximab [20]. Remission rates improve with addition of immunosuppressive agents [21, 22]. Mycophenolate mofetil and cyclophosphamide have similar outcomes [23, 24], faring better than use of cyclosporin [22], with less relapse rates. Rituximab, shown to be as effective as mycophenolate mofetil and cyclophosphamide, is often used in refractory cases [25, 26]. In combination with mycophenolate mofetil, it may allow successful tapering and withdrawal of steroid therapy [27]. Additional plasmapheresis proved non-beneficial in some case series [28, 29].

Interestingly, in treating lupus nephritis, mycophenolate mofetil has been reported as a cause of an iatrogenic-induced myopathy [30].

The different pathological features of myositis caused by anti-SRP antibodies and lupus, as well as treatment variations, suggest separate disease entities. Successful treatment of complications of both anti-SRP-associated myopathy as well as those of lupus, such as nephritis, therefore becomes difficult when both conditions occur simultaneously, as was the case with our patient.

Treatment can prove difficult as our case highlights. Both conditions respond to a variety of immunosuppressive and cytotoxic agents. The difficulty lies in finding a combination, without associated adverse events, leading to complete remission without relapse. Cyclophosphamide, ciclosporin, rituximab and mycophenolate mofetil have been used for both conditions separately with varying degrees of clinical response and remission rates. Plasmapheresis, used alone or concurrently with other agents, has also been of variable success. Our case demonstrates the benefit of plasmapheresis in treating both the patient’s anti-SRP-associated myopathy and class III focal lupus nephritis when other therapies failed to achieve success.

Identification of anti-SRP antibodies can help guide therapy in cases of difficult to treat myopathies, suggesting the need for plasmapheresis. This proved an effective strategy in our patient for whom mycophenolate mofetil with corticosteroids and rituximab infusions proved ineffective. This also induced clinical remission of the patient’s lupus nephritis. Therapy discontinuation is however associated with relapse of the clinical condition [8], as in our case. This prompts the need for continuous immunosuppressive therapy with steroid sparing agents if possible. Azathioprine had maintained control for our patient at his most recent follow-up reviews.

## References

[1] Tsokos GC, Moutsopoulos HM, Steinberg AD (1981). Muscle involvement in systemic lupus erythematosus. JAMA.

[2] Garton MJ, Isenberg DA (1997). Clinical features of lupus myositis versus idiopathic myositis: a review of 30 cases. Br J Rheumatol.

[3] Hill PA, Dwyer KM, Power DA (2000). Chronic intestinal pseudo-obstruction in systemic lupus erythematosus due to intestinal smooth muscle myopathy. Lupus.

[4] Lim KL, Abdul-Wahab R, Lowe J, Powell RJ (1994). Muscle biopsy abnormalities in systemic lupus erythematosus: correlation with clinical and laboratory parameters. Ann Rheum Dis.

[5] Suzuki S, Hayashi YK, Kuwana M, Tsuburaya R, Suzuki N, Nishino I (2012). Myopathy associated with antibodies to signal recognition particle: disease progression and neurological outcome. Arch Neurol.

[6] Valiyil R, Casciola-Rosen L, Hong G, Mammen A, Christopher-Stine L (2010). Rituximab therapy for myopathy associated with anti-signal recognition particle antibodies: a case series. Arthritis Care Res (Hoboken).

[7] Benveniste O, Drouot L, Jouen F, Charuel JL, Bloch-Queyrat C, Behin A, Amoura Z (2011). Correlation of anti-signal recognition particle autoantibody levels with creatine kinase activity in patients with necrotizing myopathy. Arthritis Rheum.

[8] Miller FW (2009). The Inflammatory Myopathies, Ch 2-Classification of Idiopathic Inflammatory Myopathies, Kagen LJ (ed.).

[9] Romisch K, Miller FW, Dobberstein B, High S (2006). Human autoantibodies against the 54 kDa protein of the signal recognition particle block function at multiple stages. Arthritis Res Ther.

[10] Miller T, Al-Lozi MT, Lopate G, Pestronk A (2002). Myopathy with antibodies to the signal recognition particle: clinical and pathological features. J Neurol Neurosurg Psychiatry.

[11] Stenzel W, Goebel HH, Aronica E (2012). Review: immune-mediated necrotizing myopathies--a heterogeneous group of diseases with specific myopathological features. Neuropathol Appl Neurobiol.

[12] Dimitri D, Andre C, Roucoules J, Hosseini H, Humbel RL, Authier FJ (2007). Myopathy associated with anti-signal recognition peptide antibodies: clinical heterogeneity contrasts with stereotyped histopathology. Muscle Nerve.

[13] Hengstman GJ, ter Laak HJ, Vree Egberts WT, Lundberg IE, Moutsopoulos HM, Vencovsky J, Doria A (2006). Anti-signal recognition particle autoantibodies: marker of a necrotising myopathy. Ann Rheum Dis.

[14] Hoogendijk JE, Amato AA, Lecky BR, Choy EH, Lundberg IE, Rose MR, Vencovsky J (2004). 119th ENMC international workshop: trial design in adult idiopathic inflammatory myopathies, with the exception of inclusion body myositis, 10-12 October 2003, Naarden, The Netherlands. Neuromuscul Disord.

[15] Whelan BR, Isenberg DA (2009). Poor response of anti-SRP-positive idiopathic immune myositis to B-cell depletion. Rheumatology (Oxford).

[16] Savey L, Bussone G, Lannuzel A, Goulvestre C, Guillevin L, Mouthon L (2012). [Necrotizing myopathy associated with anti-SRP auto-antibodies: transient efficacy of a therapeutic strategy associating plasma exchanges and rituximab]. Presse Med.

[17] Arlet JB, Dimitri D, Pagnoux C, Boyer O, Maisonobe T, Authier FJ, Bloch-Queyrat C (2006). Marked efficacy of a therapeutic strategy associating prednisone and plasma exchange followed by rituximab in two patients with refractory myopathy associated with antibodies to the signal recognition particle (SRP). Neuromuscul Disord.

[18] Yamaji K, Kim YJ, Tsuda H, Takasaki Y (2008). Long-term clinical outcomes of synchronized therapy with plasmapheresis and intravenous cyclophosphamide pulse therapy in the treatment of steroid-resistant lupus nephritis. Ther Apher Dial.

[19] Weening JJ, D'Agati VD, Schwartz MM, Seshan SV, Alpers CE, Appel GB, Balow JE (2004). The classification of glomerulonephritis in systemic lupus erythematosus revisited. J Am Soc Nephrol.

[20] Bomback AS, Appel GB (2010). Updates on the treatment of lupus nephritis. J Am Soc Nephrol.

[21] Austin HA, 3rd, Illei GG, Braun MJ, Balow JE (2009). Randomized, controlled trial of prednisone, cyclophosphamide, and cyclosporine in lupus membranous nephropathy. J Am Soc Nephrol.

[22] Cattran DC, Alexopoulos E, Heering P, Hoyer PF, Johnston A, Meyrier A, Ponticelli C (2007). Cyclosporin in idiopathic glomerular disease associated with the nephrotic syndrome: workshop recommendations. Kidney Int.

[23] Ginzler EM, Dooley MA, Aranow C, Kim MY, Buyon J, Merrill JT, Petri M (2005). Mycophenolate mofetil or intravenous cyclophosphamide for lupus nephritis. N Engl J Med.

[24] Appel GB, Contreras G, Dooley MA, Ginzler EM, Isenberg D, Jayne D, Li LS (2009). Mycophenolate mofetil versus cyclophosphamide for induction treatment of lupus nephritis. J Am Soc Nephrol.

[25] Moroni G, Raffiotta F, Trezzi B, Giglio E, Mezzina N, Del Papa N, Meroni P (2014). Rituximab vs mycophenolate and vs cyclophosphamide pulses for induction therapy of active lupus nephritis: a clinical observational study. Rheumatology (Oxford).

[26] Weidenbusch M, Rommele C, Schrottle A, Anders HJ (2013). Beyond the LUNAR trial. Efficacy of rituximab in refractory lupus nephritis. Nephrol Dial Transplant.

[27] Pepper R, Griffith M, Kirwan C, Levy J, Taube D, Pusey C, Lightstone L (2009). Rituximab is an effective treatment for lupus nephritis and allows a reduction in maintenance steroids. Nephrol Dial Transplant.

[28] Lewis EJ, Hunsicker LG, Lan SP, Rohde RD, Lachin JM (1992). A controlled trial of plasmapheresis therapy in severe lupus nephritis. The Lupus Nephritis Collaborative Study Group. N Engl J Med.

[29] Euler HH, Schroeder JO, Zeuner RA, Teske E (1991). A randomized trial of plasmapheresis and subsequent pulse cyclophosphamide in severe lupus: design of the LPSG trial. Int J Artif Organs.

[30] Galindo M, Cabello A, Joven B, Alonso A, Carreira P, Porta J, Ricoy JR (2005). Mycophenolate mofetil induced myopathy in a patient with lupus nephritis. J Rheumatol.

